# Further insights into maternal and paternal human histories in southern Iberia

**DOI:** 10.1017/ehs.2025.10006

**Published:** 2025-07-07

**Authors:** Marina González-Barrio, Luis J. Sánchez-Martínez, Rosario Calderón, Candela L. Hernández

**Affiliations:** Departamento de Biodiversidad, Ecología y Evolución, Facultad de Ciencias Biológicas, Universidad Complutense de Madrid, Ciudad Universitaria, 28040, Madrid, Spain

**Keywords:** mitochondrial genome, Y chromosome, haplogroups, population ancestry, sex-biased gene flow

## Abstract

Human genetic structure of Iberian populations has been thoroughly explored in the last decades. The internal diversity of the Iberian Peninsula becomes visible by the different phylogeographic origins of particular mitochondrial DNA and Y chromosome lineages, which show a high degree of population specificity. In the present study, we combined information on matrilineal and patrilineal variation patterns in two autochthonous populations from Andalusia region (southern Spain). A special focus is made to a male sample set where both uniparental data are available. Gene diversities estimates yielded not statistically significant differences between both types of samples and markers. Genetic ancestry among Andalusians seems to be constituted by three foremost continental origins: European, African, and Middle Eastern. The examined male group has revealed a noticeable proportion of individuals (over 45%) with a non-correspondence between maternal and paternal haplogroup origins, a signal of different population demographic histories linked to both sexes in the past. Andalusian males seem to be well differentiated according to ancestries. As expected, mtDNA diversity was much higher than that for the Y chromosome, a fact that can be caused by patrilocality, which leads to particular social structures with effects on haploid genomes in modern human populations.

## Graphical abstract

**Figure:**
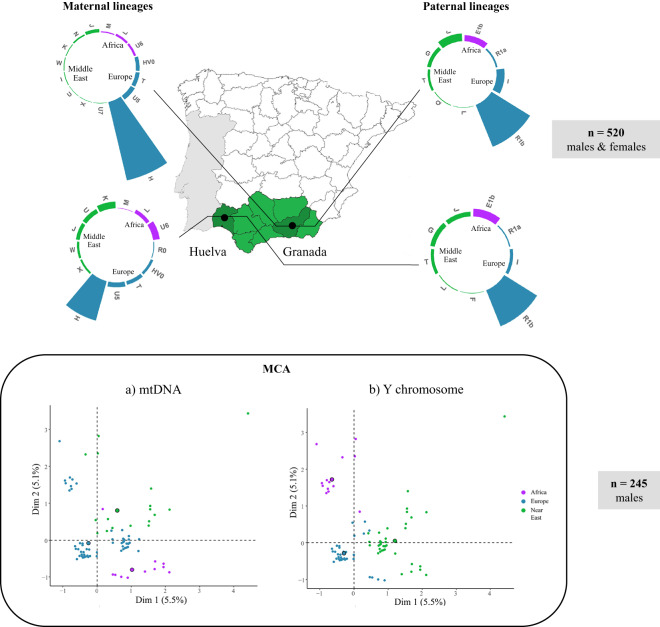

## 1. Introduction

Since ancient times, the Iberian Peninsula has been a gravitational pole of numerous and recurrent migrations associated with the melting of cultures and people from diverse origins, mainly from the Mediterranean, a geographical space of great historical significance. Quantifying the extent of genetic contributions from prehistoric and historic migration flows towards Iberia and the effects of complex internal movements –of both native and mixed populations– on the genomic compositions of modern Iberian autochthonous populations is far from easy.

Present-day understanding of the genetic structure of the populations from the Iberian Peninsula is based on classical alleles (blood groups, proteins, and erythrocyte enzymes), DNA polymorphisms, and more recently, using high-density panels of single-nucleotide polymorphisms (SNPs) (genome-wide, GW data). The latter approaches are also providing, with a high level of resolution, demographic pasts, admixture histories, and ancestral components linked to well-defined continental human groups, such as Iberians, with distinctive genomic backgrounds (Álvarez et al., 2010; Álvarez et al., 2014; Bycroft et al., 2019; Calderón et al., 2019; Font-Porterias et al., 2022; Fortes-Lima et al., 2014; González-Fortes et al., 2019; Hernández et al., 2014, 2020; Martínez-Cruz et al., 2012; Pereira et al., 2010; Pimenta et al., 2017; Rey-González et al., 2017; Santos et al., 2014; Silva et al., 2021, among others). The complex history of Iberia, including prehistorical and historical migration events, types of migrations involving males and/or females, settlement patterns, geography, cultural habits and linguistic traits, mating systems and marriage behaviours (e.g. patrilocality) have been used as key factors influencing its gene pool. The existence of an internal structure across the Iberian Peninsula, which is associated mainly with differential African genomic contributions, has also been shown, with Andalusia and the Iberian Atlantic façade being the two peninsular regions with the highest African signatures. In the case of mainland Spain (504,782 km^2^), evidence shows an increase in genetic diversity from the Cantabrian Cornice and Pyrenees Mountains in the north to the extensive Andalusian region (covering 87,268 km^2^) in the south. Similarly, genetic differentiation patterns between the eastern Mediterranean coastal area and the Atlantic slope have also been reported.

The observed diversity within Iberia becomes visible, among other means, through the different phylogeographic origins of particular mitochondrial DNA (mtDNA) and/or Y chromosome (Y-C) haplogroups (lineages). mtDNA and the non-recombining portion of the Y-C continue to be critical genomic regions contributing to the knowledge of contemporary human genetic diversity. With their particular modes of inheritance, matrilineal and patrilineal markers show a greater degree of population specificity than other genetic polymorphisms in the human genome; hence, they are particularly useful for reconstructing maternal and paternal demographic histories and tracing the phylogeographic origins of single individuals and populations (Badro et al., 2013; Kayser, 2003; Seielstad et al., 1998; Stefflova et al., 2011; Underhill & Kivisild, 2007, among others).

One of the pillars supporting modern human population genetic studies has been to explore jointly maternal and paternal genetic lineage patterns to infer ancestry, which can play a key role in shaping the genomic diversity of present-day human populations. In any population, individuals differ in their ancestry, with varying contributions from different source populations. But what is ancestry? In a genetic context, ancestry refers to the subset of paths through which the genomic material of an individual has been inherited from ancestors, likely from different geographical origins (Mathieson et al., 2020). Individuals or populations sharing ancestry or contact may be a testimony to their genetic similarities, although this assumption becomes less clear when finer-scale structural procedures are used.

Previously, mtDNA and Y-C data have been published on autochthonous Andalusian populations from the east and west, represented by the territories of Granada and Huelva, respectively (Ambrosio et al., 2010a, 2010b, 2012; Hernández et al., 2014, 2015, 2017, 2019). Some relevant results of these surveys include the presence of wide, heterogeneous, and significantly structured mtDNA lineages across Andalusia. This structure is not as conclusive for Y-C markers. Furthermore, the African influence among Andalusians is more relevant for the maternal than the paternal markers, and that impact is much stronger in the western-most territory of the region (Huelva province). The preservation of original mutational events in genetic lineages over generations has permitted the contrast of asymmetric contributions of mtDNA and Y-C lineages to the ancestral patrimony of host populations, which have settled in specific territories since ancient times. Human migrations throughout history have proceeded differently for the two sexes, largely encouraged by patrilocal social structures, which influence the movement of women from one population to another. Consequently, female migration as opposed to male migration and subsequent gene flow presumably increased the heterogeneity of geographical origins, with effects on geographic patterns of human mtDNA against Y-C distributions (Hammer et al., 2008; Heyer et al., 2012).

The present study represents a reappraisal of mtDNA and Y-C variation patterns in eastern and western Andalusia. This approach, which has never been jointly analysed, can provide more insight into the genetic history of this southern Iberian (Spanish) population. Phylogeographic structure with ancestry assignments was explored in a global sample, including males and females as well as in the subset of males with family origins in both of the Andalusian territories studied, and for which both Y-C and mtDNA data were available for each individual. In this context, male samples may add much to what can be inferred from entire samples. Scenarios of possible sex-specific behaviours that have determined the current distribution of mtDNA and Y-C haplogroups (lineages) in the region are also examined. For comparative purposes, we consider published uniparental data from other Iberian and non-Iberian Mediterranean populations.

## 2. Methods

### 2.1. Populations, samples and sampling locations

**Figure 1.**
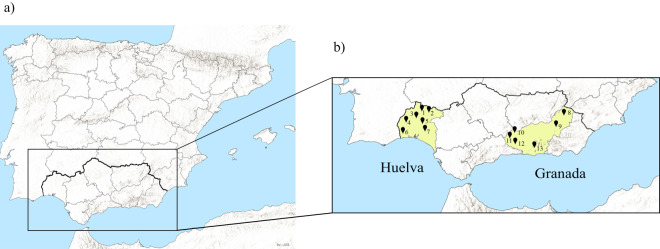
(a) The Iberian Peninsula. (b) The Andalusian region with the target provinces and the sampled municipalities. Huelva province: 1, El Repilado; 2, Aracena; 3, El Cerro del Andévalo; 4, La Puebla de Guzmán; 5, Valverde del Camino; 6, Villablanca; 7, Niebla. Granada province: 8, Huéscar; 9, Baza; 10, Montefrío; 11, Loja; 12, Alhama de Granada; 13, Órgiva.

The province of Huelva, on the western-most side of the Andalusian region, and the province of Granada, located at the eastern end of Andalusia, have been and still are the target territories selected by this team to investigate human genetic diversity patterns in southern Spain in the context of the Iberian Peninsula and the western Mediterranean (Figure 1a).

The sampling process was performed between 2004 and 2008 and involved fieldwork conducted in 13 localities (municipalities) belonging to Huelva province (sampling locations shown in Figure 1b). Outside that time period, no new biological samples have been gathered by this team from healthy autochthonous Andalusians.

The Bioethics Committee of the Carlos III Clinical Hospital in Madrid and the Complutense University of Madrid (UCM) approved the research protocols used for this study (Ref. 14/415-E_BS). Professor Calderón was the Principal Investigator (PI) of the project. All subjects (donors) provided signed informed consent for sample collection. Each donor was informed of the main scientific goals of the study, and kindly asked to provide the geographic origin of his/her family (parents and grandparents). Further sampling strategies, sample collection, and details on the history and historical demography of both studied Andalusian subpopulations can be found elsewhere (Calderón et al., 2006; Hernández et al., 2014).

A number of autochthonous individuals resulting from a randomized selection from the global collected sample set were previously analysed for uniparental molecular markers [Huelva: mtDNA, *n* = 222 (64.34%), Y-C, *n* = 167 (91.26%); Granada: mtDNA, *n* = 298 (51%), Y-C, *n* = 223 (76.00%)], and the corresponding results have been published by this research group (see references over the text). From this sample set, information on mtDNA and Y-C variation for 245 Andalusian males (121 from Huelva and 124 from Granada) is available.

### 2.2. Laboratory molecular analyses

The experimental analyses of uniparental markers and autosomal Andalusian variation are detailed in several studies (Hernández et al., 2014, 2019, 2020). The characterization of Y-C haplogroups/subhaplogroups was conducted by testing a set of 57 Y-C SNPs spread over the great clades of the Y-C phylogenetic tree. Nomenclature was mostly based on the consensus ISOGG Y-DNA Haplogroup Tree 2017 (International Society of Genetic Genealogy, http://www.isogg.org/tree/). The paternal haplogroups detected among autochthonous Andalusian samples were: BT (SRY 10,831.1) E (E-M96), F (F-M213), I (I-M170), J (J-12f2.1), G (M-201), F (F-M213), L1 (L-M22), Q (M-242), R and subhaplogroups R1* (R-M173*) and T1a (T-M70).

For mtDNA analysis, first, the hypervariable region I (HVS-I) and part of HVS-II (≈820 bp) were amplified and then 47 coding region-informative SNPs were surveyed for haplogroup assignment either by sequencing or by polymerase chain reaction–restriction fragment length polymorphisms (PCR-RFLPs; see Hernández et al., 2014). The types of maternal haplogroups detected among autochthonous Andalusian samples were R0, HV, H. J, T, U (xU6), K, NR*, N1, N2, X, U6, M, and L (Hernández et al., 2014).

In addition, GW data in 35 samples, both from Huelva and Granada, are presented in Hernández et al. (2020). The raw data genotyped consisted of 2,372,784 SNP markers with a mean genotyping rate of 99.5%. Methodologies used for evaluating biogeographic ancestry patterns and proportions, demographic histories, and admixture dynamics of that metapopulation are thoroughly described in the above-mentioned publication.

### 2.3. Uniparental inherited markers in Andalusia

Phylogeographic characteristics associated with mtDNA and Y-C markers were inferred either from haplogroups or, in some cases, from informative subhaplogroup data following Underhill and Kivisild (2007), Karafet et al. (2008), van Oven and Kayser (2009), and Pala et al. (2012). Therefore, we set up three potential geographic origins for those lineages/sublineages detected among the Andalusian individuals analysed: mtDNA haplogroups [Europe (H, T, U5, and R0), Africa (U6 and M1, North Africa) and (L1, L2 and L3, central-western Africa), and the Middle East/eastern Mediterranean (N, W, J, U7, U2, U3, U4, K, and X)]; Y-C haplogroups [Europe (R1a-M17, R1b-M269, I-M170, I1-M253, and I2-P215), Africa (E1b-M35), and the Middle East/eastern Mediterranean (F-M213, G-M201, G2-P287, J-12f2, J1-M267, J2-M172, L-M22, Q-M242, and T-M70)].

### 2.4. Statistical and genetic data analysis

Comparative analyses were first performed by considering the two Andalusian provincial territories, Granada and Huelva. Initially, maternal/paternal ancestral components were contextualized with autosomal GW data from the same southern Iberian populations. The ancestral proportions inferred from GW data were based on a global ancestry analysis performed by means of the ADMIXTURE software (Alexander et al., 2009) considering *K* = 4 clusters, as shown in Hernández et al. (2020). That test allowed distinguishing European, Middle Eastern, North African, and sub-Saharan African ancestries (see Hernández et al., 2020, p. 1045). Uniparental haplogroup compositions and assigned ancestries detected in both Andalusian samples were plotted graphically using circular bar plots constructed in the R program (R Core Team, 2023).

Moreover, the geographic distributions of the three inferred ancestral components were represented by frequency interpolation maps (ArcGIS Pro). Furthermore, PASSaGE v.2 (Rosenberg & Anderson, 2011) was used to accomplish spatial autocorrelation analysis. In this regard, Moran’s *I* autocorrelation indices were assessed to test whether European, African, and Middle Eastern lineage proportions exhibited specific structures in relation to geographic distances. Similarly, to investigate the influence of longitude and latitude on the distribution of ancestral components, a Bayesian mixed effects model was executed through the integrated nested Laplace approximation (INLA) using the R-*INLA* package (Lindgren & Rue, 2015). This procedure relies upon Bayesian inference to model the posterior marginal distribution of the parameters. Municipality altitude was set as a random effect of type ‘Random Walk’ of order 1 as follows:

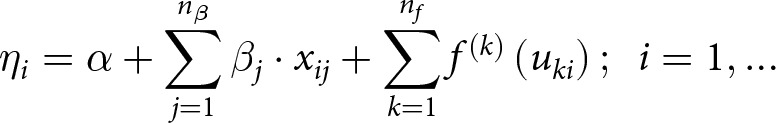


where *η_i_* is the linear predictor, α is the intercept, *β_j_ x_ij_* are the covariate parameters and covariates, and *f*^(*k*)^ are the random effects terms on some covariates.

Genetic diversity estimates based on haplogroup data from both uniparental loci were calculated using ARLEQUIN v. 3.5 (Excoffier & Lischer, 2011). Likewise, Andalusian males with information for both mtDNA and Y-C genomic markers were selected, and a bivariate exploratory analysis through contingency tables was performed to determine whether maternal and paternal ancestral sources corresponded. Afterwards, a multivariate correspondence analysis (MCA) was done to test the expected consistency using the R-*MASS* package (Venables & Ripley, 2002). This statistical analysis, which is based on a simple correspondence procedure, allows us to predict and represent relationships between categorical variables as well as to potentially identify individuals from the same groups using colour legends (Abdi & Valentin, 2007).

To examine whether the contributions of genetic ancestry observed in other surrounding human populations showed similar patterns to those found among Andalusians, we compiled data on mtDNA and Y-C variation in other Iberian/Mediterranean global population samples from the literature (Bekada et al., 2013; Boattini et al., 2013; Martínez-Cruz et al., 2012; Santos et al., 2014). Similarly, haplogroups were assigned to potential ancestries or phylogeographic origins following the information provided above. DNA samples and a summary of statistical and genetic data analysis used in the present work are provided in Table S1.

## 3. Results

**Figure 2.**
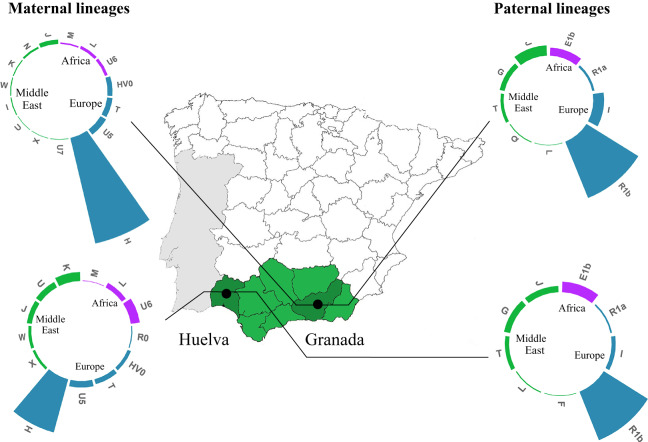
Maternal and paternal haplogroup variation patterns in Huelva and Granada provinces (Andalusian region; data from Ambrosio et al., 2010b; Ambrosio, 2011; Hernández et al., 2014).

The mitochondrial DNA and Y-C profiles of the main haplogroups and subhaplogroups observed in the entire analysed sample (*n* = 520 individuals) from the western (Huelva province) and eastern (Granada province) Andalusia slopes are illustrated in Figure 2 (further details in Table S2). Interestingly, Granada Andalusians are distinguished by a high proportion of the major European Hg H (79.53%). This haplogroup was present at a significantly lower frequency (52.70%) (*p* value < 0.05) in western Andalusians. The proportions and variations of maternal lineages originating in the Middle East across this southern Spanish region are more than visible, although important dissimilarities between the eastern and western subregions were detected [7.38% (E. Andalusia) versus 22.97% (W. Andalusia), *p* value < 0.05], with mtDNA haplogroups K1 (7.21%), J2 (3.60%), and J1 (2.35%) being the most prevalent in the region. Maternal African legacy among Andalusians has been identified through the U6a, U6b, U6c, L1b, L2a, L2b, and M1 lineages, with significant differences between the eastern (5.03%) and western (13.06%) side (*p* value < 0.05). The presence of paternal lineages from the Middle East in Andalusia is noteworthy (approximately 21% on average) and clearly dependent on geographical location within the region. Hg E1b-M35, a subclade belonging to the African haplogroup E, was observed at informative frequencies (≥5%), although signals of African Y chromosomes were relatively lower among eastern Andalusians (8%) than that among their western neighbours from Huelva (12%).

**Figure 3.**
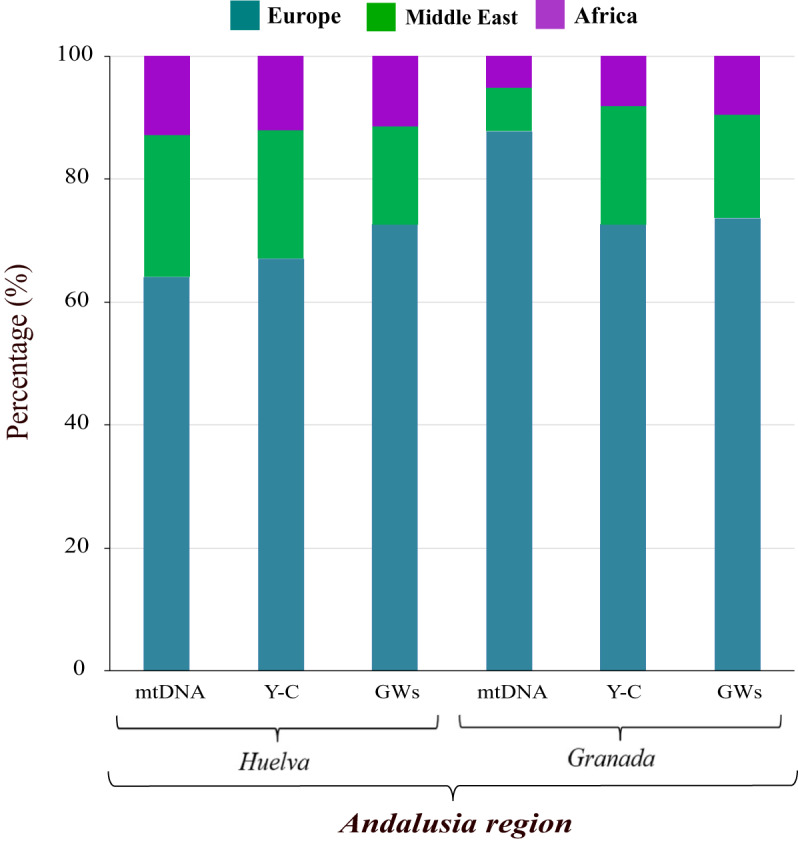
Biogeographic ancestry proportions in southern Spain (Andalusia region) determined by uniparental markers (mtDNA and Y-C) and genome-wide (GW) data.

A global representation of continental genetic ancestry proportions inferred from mtDNA, Y-C, and GW autosomal data (Hernández et al., 2020) in the studied Andalusian subpopulations is displayed in Figure 3. As can be graphically observed, European ancestry is the predominant component followed by that of the Middle East and Africa. Comparatively, Granada Andalusians are distinguished by having a much higher proportion of European genomic background. When attention is paid to ancestry population proportions, their corresponding 95% confidence intervals (CI) (see Table S3) is noteworthy, pointing out that CI estimates for mtDNA are not overlapping for any of ancestries between eastern and western Andalusians. The opposite scenario is found for Y-C and GW data. These results capture quite well the fact that differences between Granada and Huelva autochthonous people, in terms of ancestry proportions, are basically of maternal origin.

**Figure 4.**
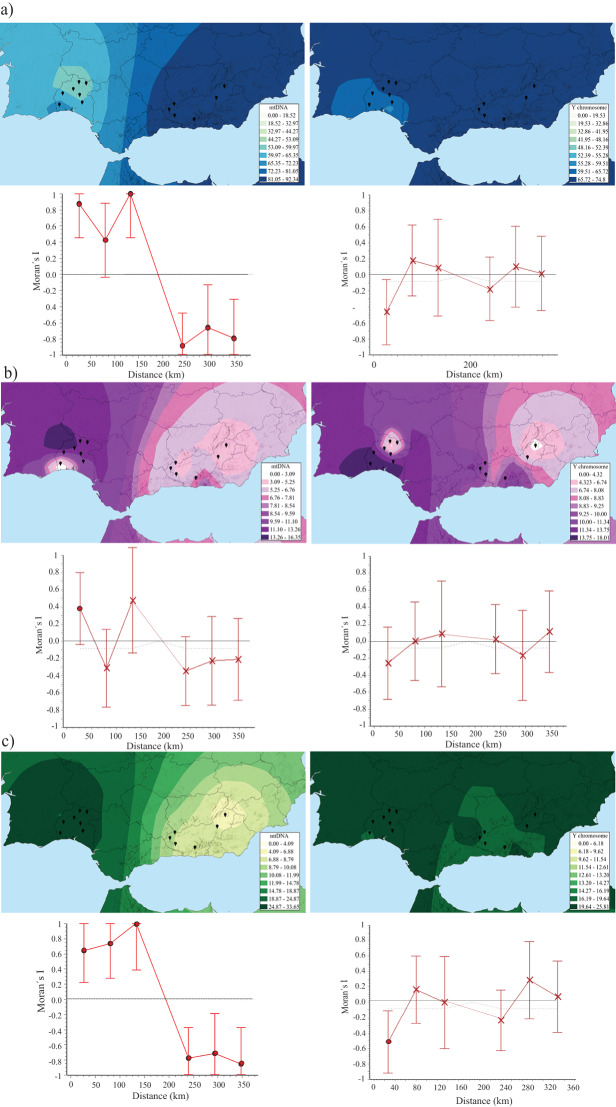
Surface interpolation maps of maternal (left side) and paternal (right side) lineages according to ancestry continental assignments [(a) European; (b) African; (c) Middle Eastern]. Spatial autocorrelation analyses are shown. Significant results of Moran’s *I* correlograms (*p* values < 0.05) are highlighted in filled red circles.

The spatial distribution patterns of both maternal and paternal lineages in relation to ancestry assignments across Andalusia are shown in Figure 4. Use of the inverse distance weight (IDW) interpolation method revealed that the European mtDNA legacy fits well with a solid eastwards-increasing gradient (Figure 4.a.1). Moran’s *I* values indicated significant correlations for both short and long geographic distances (*p* value < 0.001). Regarding African maternal haplogroups, it is interesting to highlight how Huelva, the western-most territory of Andalusia, stands out as a hotspot owing to the relatively high proportion of African mitochondrial heritage (Figure 4.b.1). The largest positive Moran’s value (*I* = 0.382 ± 0.21, *p* value < 0.05) was only perceived at the shortest distances (0–50 km). A similar scenario is observed when analysing the geographic variations in Middle Eastern maternal lineages, which revealed a gradual increase towards the Atlantic coasts of the Peninsula, with values of up to approximately 34% in Huelva Andalusians and the bordering territory of southern Portugal (Moran’s *I* coefficients *p* values < 0.01; Figure 4.c.1). Strikingly, comparable scenarios for Y-C variation were not visualized in the region. The correlograms produced from surface maps did not yield significant Moran’s values. Most of the relationships between populations were positively correlated and encompassed rather stable oscillations around zero (see Figures 4.a.2, 4.b.2, and 4.c.2).

**Figure 5.**
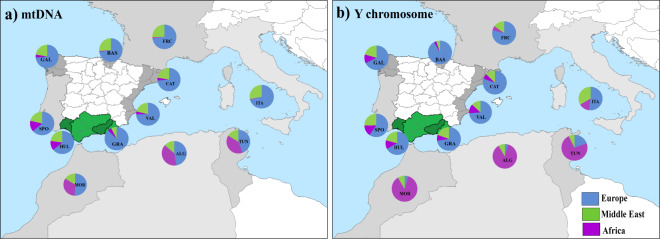
A global perspective of maternal (a) and paternal (b) continental ancestry proportions in Mediterranean populations. Iberian Peninsula: Galicia (GAL), Basques (BAS), Catalonia (CAT), Valencia (VAL), Granada (GRA), Huelva (HUL), south Portugal (SPO); other north Mediterranean areas: France (FRC) and Italy (ITA), and North Africa (Maghreb): Morocco (MOR), Algeria (ALG) and Tunisia (TUN). References in Table S4.

Human genetic studies dealing with variations in haploid genomes within the Iberian Peninsula together with other published data from non-Iberian Mediterranean populations (Figure 5, Table S4) have been used to assess how genetic ancestry components shape current genetic compositions. Figure 5a shows that mitochondrial European haplogroups are predominant in the Mediterranean, a distinction also strongly exhibited by Maghrebi populations [Morocco (50.65%), Algeria (46.79%), and Tunisia (43.84%)] (Bekada et al., 2013). Maternal patrimony from the Middle East in mainland Iberians presents a rather uniform pattern (21–27%), including the Basques (Martínez-Cruz et al., 2012). This scenario is also shared by Mediterranean neighbours [e.g. Italy (Boattini et al., 2013) and North Africa]. The exception is found, surprisingly, in the Andalusians from Granada (7.38%), which are in opposition with the high influence of the Y-C (19.38%) of similar origins among its autochthonous population. In addition, North African populations point towards a substantial prevalence of Y-C lineages of African ancestry (Morocco, 86%; Algeria, 58.32%; and Tunisia, 72.88%), with a distinct negative overall gradient in the south‒north direction (Figure 5b). This observation aligns with what is perceived along the Atlantic façade of the Iberian Peninsula, where signals of paternal African lineages are noteworthy (12–15%). Italians also share this attribute (14.30%; Boattini et al., 2013). The genomic signatures of Middle Eastern Y chromosomes are also more than perceptible in other Spanish coastal regions and populations [e.g. Galicia and Valencia (Santos et al., 2014)] and Italy (33.50%). These findings are symptomatic of the existence of ancient and diverse pathways used during Neolithic population expansion forwards through the Mediterranean.

### 3.1. Assessing genetic ancestry correspondences from mtDNA and Y chromosome lineages on autochthonous Andalusian males

A sample consisting of 245 males for which both mtDNA and Y-C data were available for each individual [121 of 183 (66.12%) from Huelva and 124 of 290 (42.76%) from Granada] was compared with the entire sample (*n* = 520, both males and females) previously investigated (see Table S5). The high mtDNA haplogroup diversity (0.9171 ± 0.0094 male sample; 0.9086 ± 0.0007 entire sample) significantly contrasts with the lower Y-chromosomal haplogroup diversity (0.6238 ± 0.0338, male sample; 0.6163 ± 0.0027, entire sample). No statistically significant differences were observed between gene diversities values emerging from mtDNA and Y-C variation when comparing both of two sample types. The reduced diversity of the Y-C with respect to mtDNA appears to be a common circumstance inherent to uniparental lineages identified in many modern human populations (Webster & Wilson Sayres, 2016), although some interesting regional variations are found worldwide that are relatively dependent on their demographic past (Lippold et al., 2014).

The weight of maternal and paternal haplogroups observed in the ‘specific’ Andalusian male sample revealed that 54.69% of males (134 of 245) carried European ancestry for both mtDNA and the Y-C. Other individuals bearing maternal European ancestry in combination with paternal markers of Middle Eastern or African origins reached frequencies of 14.69% and 8.98%, respectively. The proportion of males without mtDNA and Y-C European ancestry was approximately 8%.

**Figure 6.**
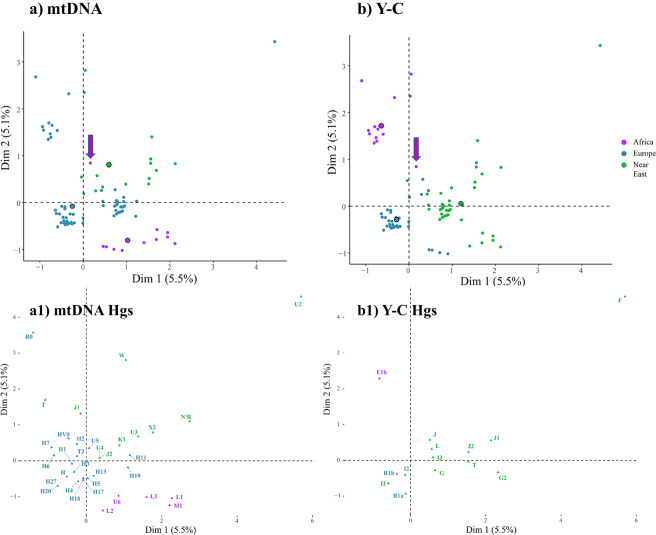
Multiple correspondence analysis (MCAs) for mtDNA (a) and Y-C (b) based on a sample set of Andalusian males. Each male individual shaping the sample carries information on both uniparental genomes. MCAs below show maternal (a1) and paternal (b1) haplogroups that have shaped the topology of individuals spread over the bidimensional space (displayed when possible). Arrows, in purple, indicate the only individual that harbours both maternal and paternal African haplogroups. Centroids are denoted by circles in solid black lines.

To further investigate the links between maternal and paternal lineages in terms of phylogeography, we performed MCAs, as shown in Figure 6. The multivariate procedure, which is descriptive in nature, allows illustration by means of perceptual mappings of the impact of continental genetic ancestries (categories) on the identification of lineage distribution patterns. Andalusian males appear to be well differentiated based on their ancestry. In the MCA plot based on mitochondrial haplogroup/subhaplogroup data (Figure 6a), the vast majority of men formed three major subgroups (subclusters), all demonstrating European maternal ancestry. However, the nature of such structuring appears to be highly influenced by the phylogeography attributed to African and Middle Eastern paternal lineages, which appear more structured and intertwined and presumably mediated by gender-specific admixture processes (Figure 6b). Accordingly, Seielstad et al. (1998) noted that Y-chromosomal lineages tend to show a higher degree of population specificity and hence may be more informative for tracing population history. The largest grouping, located in the third quadrant near the centroid, is differentiated by carrying the most representative maternal and paternal lineages that distinguish European genetic patrimony, that is, Hg H (H1) and Hg R1b-M269, respectively. The other related cluster, in the second quadrant, comprises individuals who harbour European maternal lineages in combination with Y chromosomes of African origin. The lower part of the first quadrant is identified by reciprocated paternal lineages of Middle Eastern origin. One further grouping, more scattered at the bottom of the fourth quadrant, is shaped by people carrying African mtDNA lineages together with patrilineal haplogroups originating either in Europe or the Middle East (Figure 6b). Curiously, very few men appear isolated over the bidimensional space. The position of an outlier from western Andalusia on the left side of the first quadrant, should be identified, in genomic terms, as African as he carried both maternal (U6) and paternal (E1b-M35) ancestries with that continental origin. Other study cases are distinguished by sharing Middle Eastern mtDNA and African Y chromosomes.

### 4. Discussion

Phylogeographic lineages observed from both mitochondrial DNA genome and Y-C analyses have allowed us to infer structures and genetic ancestries among Andalusians, which appear to be primarily constituted from three continental origins: European, African, and Middle Eastern, a pattern not so different from other neighbouring Mediterranean populations. The maternal haplogroup H (H1 subhaplogroup) and the paternal R1b-M269 are markers for western Europe. Similarly, the African influence on the Andalusian genomes is recognized by the presence of the mtDNA North African haplogroups U6 and M1 and the west-central African haplogroups L1 and L2 as well as the paternal E-M81, a branch identified within the E1b-M35 clade. The E-M81 marker is associated with the Berber component of Islamic expansion. The contributions of the Middle Eastern gene pool and other neighbouring populations of the eastern Mediterranean to Iberia have also been shown to be relevant. All these observations are congruent with historical and archaeological evidence from the Peninsula in general, and the region of Andalusia in particular.

African ancestry within Iberia has been widely investigated. In the frame of the European continent, it is well known that the highest African genetic signatures are concentrated in southern Iberia and the Iberian Atlantic façade (Ambrosio, 2011; Hernández et al., 2014; Pereira et al., 2005; Santos et al., 2014). The area west of the Mediterranean with the Strait of Gibraltar and its surrounding coasts represents one of the main migration corridors (it is the shortest) for the rather highly directional entry of human populations in the south–north direction and reciprocally. The presence of the European component is typically large in north-western African native populations. This pattern is illustrated by maternal subhaplogroup H1, which has frequencies (40–45%) comparable to those reported in south-western Europe (Currat et al., 2010; Hernández et al., 2017).

Ancient migrations across the Strait of Gibraltar to the Iberian Peninsula appear to have occurred since the Holocene (∼11,000 BP) and have been continuous over time. These human movements have been confirmed via specific mitogenome analyses and massive SNP genotyping procedures (Cerezo et al., 2012; Fregel et al., 2018; González-Fortes et al., 2019; Hernández et al., 2015, 2020; Simões et al., 2023). However, human mobility from North Africa to southern Europe was particularly intense during the Muslim occupation (711–1492) of the Iberian Peninsula. The North African population of Berber origin was the largest population that reached Iberia at the beginning of the long-lasting Muslim presence on the Peninsula. The proportion of Arab people, however, was relatively small at 1.5–2% and 0.4–5% of the population of the Peninsula at that time (Guichard, 1976). The genetic effects of those migrants, mostly male soldiers of reproductive age and unaccompanied by their families in the early phases of the process, would have led often to visible population sex-specific admixture in terms of introgressions of African male-inherited Y chromosomes into native Iberian populations. The number of North African female migrants accompanying spouses who reached Iberia with family members or the number of single women who migrated there while the occupation lasted is unknown. In this context, additional genetic effects could also be strengthened by polygyny, a form of marital behaviour mainly adopted by wealthy social groups (e.g. Arab people) living in cities. Polygamy (polygyny) is a type of marriage involving a man (husband) with several wives who are not necessarily native to their territories of origin (endogamous and exogamous marriages), thus encouraging the dispersal of women and the subsequent spread and transmission of mtDNA lineages.

The rather low signals of maternal African ancestry in Granada Andalusians (present study) may be a remarkable, unexpected finding. The Kingdom of Granada or *Nazari Kingdom* represented the last territory of Muslim occupation within the Iberian Peninsula. The expansion of the Christian *Reconquista* in response to the Muslim invasion was completed in 1264, with the exception of Granada, which was reconquered in 1492. The consequences of the *Reconquista* of Granada and surrounding areas were, among others, that Arabs, Berbers, and Jews were expelled from these areas and replaced largely by old Christian farmers from different Spanish regions [i.e. north-western (Galicia), northern Meseta (Castille and León), and north-eastern (Aragón)] and even Portugal. This planned internal human mobility associated with well-connected routes to southern Spain led to multiple complex episodes of colonization, with new settlements and intermarriages between new migrants and native Andalusians, as would have been the norm. The impact of these episodes leads to different structures of genetic variation. In this context, human leukocyte antigen (HLA) genes and haplotypes can represent a case study. At ‘*The Alpujarras*’, a historical Andalusian small region (*Comarca*) located between the provinces of Granada and Almeria, Arnaiz-Villena et al. (2022) found that the most frequent HLA haplotypes among the autochthonous people correspond with a European origin, typically from north-western and central Spain. Our findings here, and others previously published by our research group, closely parallel these observations.

Genetic studies have suggested that at least a quarter of the human maternal lineages currently distributed across Europe originated in the Middle East. The signal is also significant for the Y-C on the continent (Botigué et al., 2013). It is likely that a substantial portion of the Middle Eastern Mediterranean ancestry present in the Iberian Peninsula (e.g. coastal Iberian populations) is associated with Neolithic expansions and not before. An earlier survey aimed to assess the diversity patterns of J and E patrilineal haplogroups/subhaplogroups in western and eastern Andalusians, the coalescent time estimates from intrahaplogroup-associated Y-STR haplotype diversity data do not support the introduction of lineages to the Iberian Peninsula before the arrival of Neolithic agriculture (Ambrosio et al., 2010b). In both studied Andalusian subpopulations, the paternal J2 (M-172) was the most highly represented (∼5%) of all the J subhaplogroups observed. Y-C Hg J is one of the most common markers in the current Middle Eastern and other neighbouring eastern Mediterranean populations.

The presence of Jews in the Iberian Peninsula appears to date long before the Roman Empire. However, the number of Jews that entered Spain is unknown, although it is assumed that the demographic size of this group increased noticeably during the Muslim expansion (Caro Baroja, 1962). Similar to the Berbers and Arabs, Jews in Spain who did not convert to Christianity were expelled in 1492, and a large proportion moved to Portugal and south-east Europe. However, it is important to note that the real number of Jews driven from Spain is unknown. The Jews who are relevant for assessing their signature in Iberian genomes would be those who remained in Spain whose descendants still live in the country and whose ancestor origins are known.

Other genetic influences on Andalusian genomes could also come from proto- and early historical civilizations, such as Phoenicians, Greeks, and Tartessians, who occupied south-western Iberia, with its coastal and internal territories, during the first millennium BC (Dietler & López-Ruiz, 2009; Fernandez-Jurado et al., 1997; López-Ruiz & Celestino Pérez, 2016; Tsirkin, 1997). For example, the Tartessian civilization of south-west Iberia developed intense contacts with eastern Mediterranean populations (i.e. the Levant and surrounding geographical areas). Two important discoveries in Spain are worth noting, as they provide important insights into peninsular protohistory. Particularly relevant is the great Tartessian necropolis known as ‘La Joya’ (second half of the eighth century to 550 BC; Torres-Ortiz, 1999), which is located in estern Andalusia (Huelva). The other, known as ‘Casas del Turuñuelo’ (VIII–IV BC; Rodríguez González & Celestino Pérez, 2019), is found in Extremadura, the south-western Spanish region adjacent to the western slope of Andalusia. Both necropolises, rich in archaeological remains, are referential to Tartessian cultural practices as well as for understanding facets of the peopling of the Iberian Peninsula. Our survey revealed that the highest maternal genetic contributions from the Middle East are observed in western Andalusia. The J2 subhaplogroup appears to be related to the Greek and Phoenician colonies that were established in the Iberian Peninsula, because maritime trade is linked mainly to the significant metal resources in southern Iberia.

The availability of genomic data from mtDNA and Y chromosomes in our male group has revealed interesting particularities. First, a high proportion of individuals showed non-correspondence between maternal and paternal haplogroup origins (45.31%, 111 of 245). These results should be explained as indicators of different population demographic histories linked to both sexes in the past. Second, as expected, mitochondrial genome diversity was much greater than that for the Y-C, possibly because of different potential factors, including patrilocality, which is an expression of sex-biased mobility in that the woman moves into her husband’s natal household or residence place after marriage (Cavalli-Sforza & Bodmer, 1971). This cultural practice is typical in most modern societies (≈70%) (Burton et al., 1996). In contrast, matrilocality, in which the man moves to his wife’s birthplace or residence place after marriage, is practised only in certain ethnic or social groups. These current phenomena on marital residence patterns have had a great influence in shaping modern genetic diversity (Jobling et al., 2014, pp. 155–156). In this line, Oota et al. (2001) analysed mtDNA and Y-C variation in three patrilocal and three matrilocal groups among the hill tribes of northern Thailand. The authors observed that the haplotype diversity for mtDNA (HVS-I region) was higher in all patrilocal groups (0.937, as average) than in any matrilocal groups (0.860, as average). Nevertheless, they found an opposite scenario when comparing the Y-STR haplotype diversity (0.863: patrilocal groups; 0.965: matrilocal groups). Subsequent published surveys with similar focuses have shown convergent results with exceptions for Y-C diversity (e.g. Gunnarsdóttir et al., 2011 and others).

Patrilocality leads to complex social marital structures with a strong impact on the low diversity of the Y-C within groups, greater between-group genetic differences than mtDNA (Oota et al., 2001; Wilkins & Marlowe, 2006), and particular genetic structures for mtDNA and the Y-C. These effects could be applied to mass migrations from northern Africa entering the Iberian Peninsula in the Middle Ages, where natives would have brought deep cultural packages, including patrilocality and/or polygynous habits, the latter associated with biased male reproductive success and long-standing family histories of close consanguinity (e.g. first cousin unions preferentially; Ennafaa et al., 2011; Nothnagel et al., 2010). Thus, differences between populations caused by patrilocality would be significantly female biased. The phenomenon of patrilocality, the most common pattern in human agricultural societies, is practised indistinctly in both unrelated and biologically related couples (Calderón et al., 2018).

Overall, continuing to explore the genetic heritage of Iberian populations should be pursued because of the significant contribution of these investigations to understanding current paternal and maternal population histories with respect to historical demographic movements. Importantly, the reliability of an ancestry assignment depends on the availability of updated population databases to compare the obtained results and their continental distributions. Therefore, further analyses of coastal and inland Iberian populations are needed; in particular, studies of those living in understudied geographical regions.

## Supporting information

González-Barrio et al. supplementary material González-Barrio et al. supplementary material

## Supplementary material

The supplementary material for this article can be found at https://doi.org/10.1017/ehs.2025.10006.

## Research transparency and reproducibility interest

All data used in this study are provided within the manuscript, the supplementary material or previous studies from our group.
