# Supplementary material for: Further insights into maternal and paternal human histories in southern Iberia
Source: Evol Hum Sci. 2025 Jul 7;7:e22. doi: 10.1017/ehs.2025.10006 (PMC12344598; doi:10.1017/ehs.2025.10006)
Supplement: González-Barrio et al. supplementary material [file S2513843X25100066sup001.pdf]

Supplementary Material

Further insights into maternal and paternal human histories in southern Iberia

Marina González-Barrio, Luis J. Sánchez-Martínez, Rosario Calderón, Candela L. Hernández

Table S1. DNA samples and a summary of statistical methodology used in genetic data analysis. 2

Table S2. Frequencies of the identified mtDNA and Y-C haplogroups in the Andalusian sample....... 3

Table S3. Ancestry proportions and the corresponding 95% confidence intervals (CIs). 4

Table S4. Phylogeographic diversity in western and central Mediterranean populations.................... 5

Table S5. Frequencies of the identified mtDNA and Y-C haplogroups in the male sample................. 6

Table S1. DNA samples and a summary of statistical methodology used in genetic data analysis.

	**Genomic Variation**			
**Andalusian Samples**	**mtDNA**	**Y-C**	**Statistical and genetic data analysis**	**Software**
Huelva	222	167	Circular Barplots; Interpolation Maps; Spatial Autocorrelation; Bayesian Mixed Effects Model; Genetic Diversity	R program; ArcGIS Pro; PASSaGE v.2; R-INLA package; ARLEQUIN v 3.5
Granada	298	223		
**Male Andalusian Samples**	**Genomic Variation mtDNA *vs* Y-C**			
Huelva	121		Multivariate Correspondence Analysis (MCA) N= 245	R-MASS package
Granada	124			

Table S2. Frequencies of the identified mtDNA and Y-C haplogroups in the Andalusian sample.

Origin	mtDNA	N	Frec. Mt (%)
EUROPE	H*	43	8.27
	HV0	17	3.27
	H1	122	23.46
	H2	6	1.15
	H3	58	11.15
	H4	15	2.88
	H5	22	4.23
	H6	24	4.62
	H7	12	2.31
	H10	3	0.58
	H11	5	0.96
	H13	9	1.73
	H17	6	1.15
	H18	6	1.15
	H20	4	0.77
	H27	2	0.38
	R0	2	0.38
	T	4	0.77
	T2	17	3.27
	U5	26	5.00
AFRICA	U6	24	4.62
	M1	4	0.77
	L	16	3.08
MIDDLE EAST	J1	17	3.27
	J2	2	0.38
	K1	18	3.46
	K2	2	0.38
	X2	6	1.15
	W	8	1.54
	U2	2	0.38
	U3	8	1.54
	U4	3	0.58
	U7	1	0.19
	N´R	4	0.77
	I2	2	0.38
N		520	100

Origin	Y-C	N	Frec. Y-C (%)
EUROPE	R1b-M269	237	60.15
	R1a-M17	8	2.03
	I1-M253	14	3.55
	I2-P215	18	4.57
AFRICA	E1b-M35	38	9.64
MIDDLE EAST	J*-12f2	1	0.25
	J1-M267	11	2.79
	J2-M172	23	5.84
	G*-M201	1	0.25
	G2-P287	22	5.58
	T-M70	15	3.81
	L-M22	3	0.76
	F-M213	1	0.25
	Q-M242	2	0.51
N		394	100

Table S3. Ancestry proportions and the corresponding 95% confidence intervals (CIs).

	**Marker**	**European Ancestry**	**CI**	**Middle Eastern Ancestry**	**CI**	**African Ancestry**	**CI**
**HUELVA**	mtDNA	0.641	0.575-0.700	0.230	0.179-0.289	0.129	0.092-0.181
	C-Y	0.671	0.596-0.737	0.210	0.155-0.277	0.120	0.079-0.178
	GWs	0.726	0.680-0.772	0.160	0.124-0.196	0.114	0.078-0.150
**GRANADA**	mtDNA	0.878	0.833-0.908	0.071	0.049-0.109	0.051	0.031-0.081
	C-Y	0.726	0.665-0.781	0.193	0.148-0.250	0.081	0.051-0.122
	GWs	0.737	0.694-0.779	0.169	0.129-0.208	0.095	0.052-0.137

Table S4. Phylogeographic diversity in western and central Mediterranean populations. Data shown in percentages.

Populations		EUROPE		AFRICA		MIDDLE EAST		*N*	
		mtDNA	Y-C	mtDNA	Y-C	mtDNA	Y-C	mtDNA	Y-C
[1]Galicia	GAL	73.30	67.50	4.10	11.50	22.40	20.60	555	443
[2]Basque Country	BAS	72.76	91.85	0.79	3.11	26.33	5.030	881	835
[1]Catalonia	CAT	73.90	79.90	3.80	6.90	22.30	12.60	469	231
[1]Valencia	VAL	74.80	73.50	4.00	12.80	21.10	13.30	250	163
[*]E. Andal (*Granada*)	GRA	87.58	72.69	5.03	7.93	7.38	19.38	298	227
[*]W. Andal (*Huelva*)	HUL	63.96	67.07	13.06	11.98	22.97	20.96	222	167
[1]South Portugal	SPT	63.70	59.70	14.90	15.00	21.20	25.10	268	191
Iberia	IB	73.23	78.04	4.81	7.99	21.86	13.78	2943	2257
[3]France	FRC	70.39	79.00	1.70	5.94	24.72	14.96	1304	776
[4]Italy	ITA	69.00	52.30	0.81	14.30	27.60	33.50	865	884
[3]Morocco	MOR	50.65	4.60	33.41	86.00	15.72	8.56	1194	760
[3]Algeria	ALG	46.79	32.03	38.73	58.32	13.45	9.62	372	156
[3]Tunisia	TUN	43.84	19.47	38.63	72.88	16.26	7.15	591	601

[1]*Santos et al., 2014, Am J Hum Biol, 26:130-41.*
[2]*Martínez-Cruz et al., 2012, Mol Biol Evol, 29:2211-22.*
[3]*Bekada et al., 2013, PLoS One, 8:e56775.*
[4]*Boattini et al., 2013 PLoS One, 8:e65441.*
[*]*Huelva and Granada samples (current study)*

Table S5. Frequencies of the identified mtDNA and Y-C haplogroups in the male sample.

Origin	mtDNA	N	Frec. Mt (%)
EUROPE	H*	21	8.57
	HV0	11	4.49
	H1	52	21.22
	H2	4	1.63
	H3	29	11.84
	H4	3	1.22
	H5	8	3.27
	H6	16	6.53
	H7	4	1.63
	H10	3	1.22
	H11	3	1.22
	H13	3	1.22
	H17	4	1.63
	H18	4	1.63
	H20	1	0.41
	H27	2	0.82
	R0	1	0.41
	T	2	0.82
	T2	7	2.86
	U5	14	5.71
AFRICA	U6	11	4.49
	M1	3	1.22
	L	8	3.27
MIDDLE EAST	J1	8	3.27
	J2	1	0.41
	K1	12	4.90
	K2	1	0.41
	X2	2	0.82
	W	2	0.82
	U2	1	0.41
	U3	2	0.82
	U4	1	0.41
	N´R	1	0.41
N		245	100

Origin	Y-C	N	Frec. Y-C (%)
EUROPE	R1b-M269	146	59.59
	R1a-M17	3	1.22
	I1-M253	10	4.08
	I2-P215	9	3.67
AFRICA	E1b-M35	26	10.61
MIDDLE EAST	J*-12f2	1	0.41
	J1-M267	6	2.45
	J2-M172	14	5.71
	G*-M201	1	0.41
	G2-P287	13	5.31
	T-M70	12	4.90
	L-M22	2	0.82
	F-M213	1	0.41
	Q-M242	1	0.41
N		245	100
